# Biographical

**Published:** 1877-06

**Authors:** Jos. G. Cameron, E. Osmond

**Affiliations:** Cincinnati


### BIOGRAPHICAL.

C. B. CHAPMAN, M. D., of Madison, Wisconsin, died in that city of pneumonia, on May 8th 1877, in the 62nd year of his age.

Dr. Chapman was born in Middlebury, Vermont, July 7th, 1815. He settled in Madison, Wisconsin, in 1846, and at once entered upon the practice of medicine. He was a thoroughly read physician in all its branches, and was a practical surgeon with few if any superiors in the north west.

He was a man free from ostentation; modest in all things; firm in his convictions; conscientious in the discharge of every duty; actuated in all his labors by purely Christian principles. He occupied a professorship in the Miami Medical College, and in the Ohio College of Dental Surgery, both of this City. He was Professor of Anatomy, in the latter Institution for several years and filled the position with great acceptance. His ability as a teacher was of a very high order. He was also for many years engaged in teaching in literary institutions. He has traveled much in the Old World, having made two extensive trips—the first in 1851, and the second in 1875—devoting a year or more to each trip. His letters containing accounts of his travels, were of very great interest. He was highly esteemed by all who knew him. In his death the community in which he lived has lost an honored and valuable member.

Died in this city on the 24th of March, 1877, Dr. D. B. Wheeler, D. D. S.

Dr. Wheeler was born in Orange, Mass., July, 1815. He came to Cincinnati in 1839, and having had a good academic education, began the study of dentistry with Dr. John Allen about 1841, and was under his pupilage two years. He then entered the practice of his profession in this city, and continued it up to the time of his death.

In 1843 he married Miss Eliza Allen, who with three children, survives him.

Dr. W. was a successful professional man; the elements of his success were his professional ability, integrity and careful attention to his business. He enjoyed the esteem and high regard of his professional brethren wherever he was known, and not less was this true of those who were his patients.

He was a member of several dental societies, but his special effort for his chosen profession was exhibited in the great interest he took in the prosperity and success of the Ohio College of Dental Surgery. He was for many years one of its trustees, and was usually a member of the committee having charge of the property.

As a man and a citizen he was held in high esteem by the community in which he lived; he often occupied places of trust and honor, and always with the greatest acceptance.

He was a true and steadfast friend and so unsuspecting was he of the fidelity of others, that he sometimes suffered financially thereby, but even in such cases, he usually attributed it to unfortunate circumstances rather than to a want of integrity in those whom he trusted.

He was not ostentatious, he instinctively shrank from all public notoriety or display, but he was possessed by that quiet firmness in the discharge of duty, that made him very efficient in whatever he attempted to do.

He was conscientious in all that he did, and lived to bless man kind in his sphere of action.

At a meeting of the Dental profession of this city the following preamble and resolutions were passed.

"Whereas, It has pleased our heavenly Father to remove from our midst by death our professional brother, Dr. Benjamin D. Wheeler; therefore,

"Resolved, that in the death of Dr. Wheeler the profession of Cincinnati have sustained a great loss. Always solicitous for the advancement of dentistry, he was a warm advocate and supporter of associated effort for this object. He was a friend of dental education, and for a long time previous to his death had been identified with the management of the Ohio Dental College. Of a modest and unassuming disposition, he was yet ever ready to impart knowledge when asked. As a man and a citizen we are also pleased to bear testimony that he was always honorable, generous, genial and sympathetic.

"Resolved, That a copy of this resolution be transmitted to the family of the desceased, with assurance of our sympathy in their bereavement."

Jos. G. Cameron, *President.*
E. Osmond, *Secretary.*

Cincinnati, March 31, 1877.

